# Supplementary material for: Chromothripsis during telomere crisis is independent of NHEJ, and consistent with a replicative origin
Source: Genome Res. 2019 May;29(5):737–49. doi: 10.1101/gr.240705.118 (PMC6499312; doi:10.1101/gr.240705.118)
Supplement: Supplemental Material [file supp_gr.240705.118_Supplemental_file_1.zip › contigs/annotated_contigs/DB116/contig.2.DB116_length_666_mean_cov_7.87987987988.docx]

**DB116_length_666_mean_cov_7.87987987988**

```
CTTTGCTCTGATACAATGTGAGAGAGATCCCTTGTTACTCTTCTTATGTGGTACTAATTTGCCTTTCTCTCCCTATGACCAGTTCTATC
 >chr13:61742959-61743307 + E=1e-185
TATTAATTTTCTTGAGACCCAGGACTTCTGGGGCATAGGAGAGCTGGGATGAGGCTGTGCTCAAGTCTCTGATCTCTCTTCTAAGACCT

GATTACATTTCTGATAGCAGAACCCACTTTATCCAGACAGCAGAATAAGCCCAATTGGGAAGGGTGCTTAATCTTTAGTCCTCTGACTT

ATCTGTAAGACATTTTCCCTCAACAGACCTGACCTATTTTTCTACTACCTTTGAAAATAAGATCAGCTGAAATGAATGT|TA|TATATA
                                                                                >chr13:
TATATAACAATAAAAAATAATACAGCAGCTAAAACTAATACAAATAAAATACAGTGTAAAACTATTACATAGAATTCACATTGTATTAG
61752306-61752626 + E=9e-178
GTATTATAAGTAATCTAGAGATGATTTAAGGCTTACAGAAAGAAGTGCAGAGGTTGTATGTAAACACCATGCCATTTTATATAAGAAAA

TTGAGCATCTGTCAATTTTGGTATCCGTGAGGGTCCTGGAAGCAATCCCCTACCGATACAGAGGGACCAGTGTAATGGGTAGAGACCAG

GCTGGCTGCTAAATATTCTAGAATGCACAAGACAAAGAATTATCT
```
